# Mechanical Metamaterials Foams with Tunable Negative Poisson’s Ratio for Enhanced Energy Absorption and Damage Resistance

**DOI:** 10.3390/ma11101869

**Published:** 2018-10-01

**Authors:** Shaohua Cui, Baoming Gong, Qian Ding, Yongtao Sun, Fuguang Ren, Xiuguo Liu, Qun Yan, Hai Yang, Xin Wang, Bowen Song

**Affiliations:** 1 Department of Materials Science and Engineering and Tianjin Key Laboratory of Advanced Joining Technology, Tianjin University, Road Weijin 92, Tianjin 300072, China; cui_shaohua@tju.edu.cn (S.C.); gongbm@tju.edu.cn (B.G.); 2 Department of Mechanics and Tianjin Key Laboratory of Nonlinear Dynamics and Control, Tianjin University, Yaguan Road 135, Tianjin 300350, China; qding@tju.edu.cn (Q.D.); renfuguang99@163.com (F.R.); 13043280596@163.com (B.S.); 3 State Key Laboratory of Mechanical System and Vibration, Shanghai Jiao Tong University, Shanghai 200240, China; 4 State Key Laboratory for Strength and Vibration of Mechanical Structures, Xi’an Jiaotong University, Xi’an 710049, China; 5 State Key Laboratory of Structural Analysis for Industrial Equipment, Dalian University of Technology, Dalian 116023, China; 6 Key Laboratory of Aeroacoustics and Dynamics, Aircraft Strength Research Institute, Xi’an 710065, China; qunyan_ac@163.com (Q.Y.); asricyh@163.com (H.Y.); nwpu2008301165@163.com (X.W.)

**Keywords:** convex-concave foams, tunable negative Poisson’s ratio, enhanced energy absorption and damage resistance

## Abstract

Systematic and deep understanding of mechanical properties of the negative Poisson’s ratio convex-concave foams plays a very important role for their practical engineering applications. However, in the open literature, only a negative Poisson’s ratio effect of the metamaterials convex-concave foams is simply mentioned. In this paper, through the experimental and finite element methods, effects of geometrical morphology on elastic moduli, energy absorption, and damage properties of the convex-concave foams are systematically studied. Results show that negative Poisson’s ratio, energy absorption, and damage properties of the convex-concave foams could be tuned simultaneously through adjusting the chord height to span ratio of the sine-shaped cell edges. By the rational design of the negative Poisson’s ratio, when compared to the conventional open-cell foams of equal mass, convex-concave foams could have the combined advantages of relative high stiffness and strength, enhanced energy absorption and damage resistance. The research of this paper provides theoretical foundations for optimization design of the mechanical properties of the convex-concave foams and thus could facilitate their practical applications in the engineering fields.

## 1. Introduction

Metamaterials are rationally designed artificial materials whose effective properties arise not from the bulk behavior of the materials that compose them, but from their deliberate structuring [1,2,3,4]. Unusual mechanical properties of the metamaterials, such as unique negative Poisson’s ratio (NPR), superlight weight, high stiffness, strong strength, high specific energy absorption, excellent fracture toughness, and vibration reduction characteristics, play a vital role for their multifunctional applications in the fields of mechanical and aerospace engineering [5,6]. NPR foams, which get fatter when stretched and thinner when compressed [7], are a typical kind of mechanical metamaterials. When compared with the conventional foams with a positive Poisson’s ratio (PPR), the NPR foams could have the advantages, such as the ability to undergo synclastic curvature, enhanced toughness, increased indentation resistance, improved shear stiffness, sound dampening properties, self-adaptive vibrational damping, and shock absorption, etc. [8,9,10,11,12].

To explore mechanical properties of the NPR foams and thus promote their applications in the fields of mechanical and aerospace engineering, a variety of NPR foams have been proposed by researchers in the past several decades. Prof. Rod Lakes is the scientist who first proposed the idea of man-made NPR foams, i.e., the reentrant foams [13], which showed superior indentation resistance [8] and fracture toughness [14] to the conventional foams and were very suitable to be made as the polymer seat cushions [15,16]. Evans et al. [17] proposed the reentrant three-dimensional elongated dodecahedron NPR foams. The group of Prof. Scarpa systematically studied mechanical properties of the NPR polyurethane foams, which showed better dynamic crushing, damping and acoustic behaviors [18], superior tensile fatigue performance [19,20], higher dynamic stiffness, and enhanced viscous dissipations [21] than the conventional foams of comparable density. In recent years, many new kinds of NPR foams of various cell geometries have been proposed. Based on the very simple initial geometric shapes, i.e., spherical voids in a cubic matrix, Shen et al. [22] proposed a series of cubic foams with NPR over a large strain range. Through three-dimensional (3D) printing and investment casting technology, Xue et al. [23] fabricated the Al-based auxetic re-entrant foams with sound surface and interior qualities and investigated their compressive properties. Adopting the interlocking assembly concept, Wang et al. [24] introduced the interlocking assembled 3D re-entrant NPR foams. Ai and Gao [25] designed four types of 3D bi-material lattice foams with both NPR and non-positive coefficient of thermal expansion. In recent years, the groups of Prof. Gambarotta and Prof. Bacigalupo systematically studied the overall mechanical properties (e.g., equivalent elastic properties and wave propagation characteristics etc.) of several kinds of metamaterials, such as hexachiral [26,27], tetrachiral [26,28,29,30] and anti-tetrachiral [31,32,33] materials, periodic beam lattice materials [34,35,36], rigid periodic blocky materials [37], and so on.

In this paper, mechanical properties of the orthogonal isotropic NPR convex-concave foams (CCF) [38] are investigated through the experimental and finite element methods. The CCF are constructed by replacing cell edges of the conventional open cell foams (COF), whose straight cell edges of square cross sections are arranged in planes at forty-five degrees relative to each other [38], with the sine-shaped cell edges [39,40,41,42,43,44] of equal mass but different cross sections. In the open literature, only NPR effect of the CCF is simply mentioned [38]. In practice, optimization design of the mechanical properties of the CCF plays a vital role for their engineering applications. To facilitate their practical applications in the engineering fields, mechanical properties of the CCF need to be deeply and systematically understood. Therefore, in this paper, based on the polymer materials VeroWhite Plus, mainly choosing one COF and three kinds of CCF of equal mass as examples, the effects of geometrical morphology on mechanical properties of the CCF, including elastic moduli, energy absorption, and damage characteristics, are systematically investigated combining the experimental and finite element methods.

## 2. Geometrical Models of the CCF

**Figure 1:**
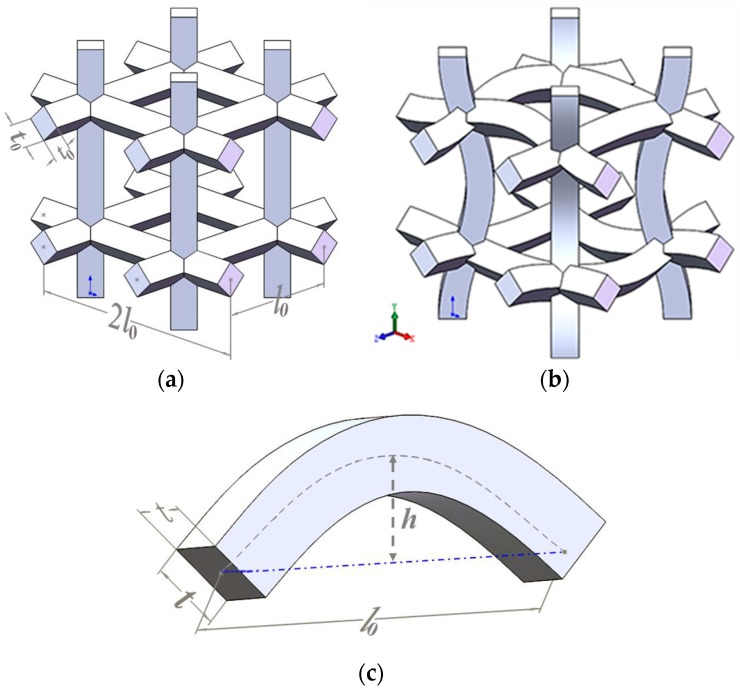
(**a**) Cubic unit cell model of the conventional open-cell foams with straight cell edges, of square cross sections, arranged in planes at forty-five degrees relative to each other, in which the edge length is $l_{0}$ and the edge thickness is $t_{0}$; (**b**) Cubic unit cell model of the negative Poisson’s ratio convex-concave foams, which are constructed by replacing cell edges of the conventional open cell foams of (**a**) with sine-shaped cell edges of equal mass but different square cross sections; (**c**) Schematic diagram of the sine-shaped cell edges of the convex-concave foams (CCF) with chord span $l_{0}$, chord height *h*, and thickness *t*.

**Figure 2:**
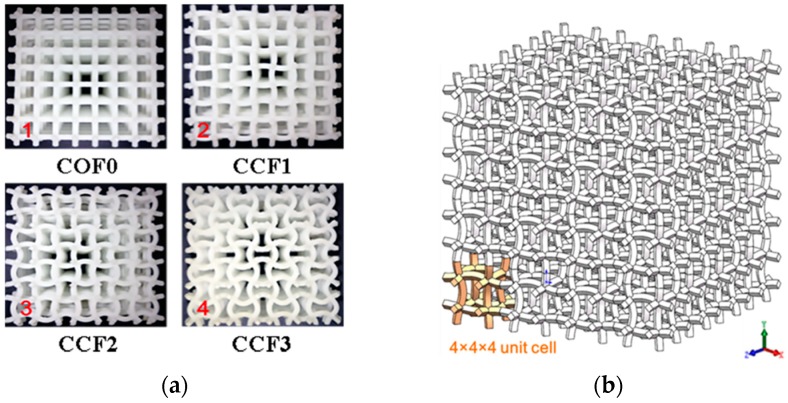
(**a**) Front views of the COF sample and the corresponding three kinds of CCF samples of equal mass: (**a1**) COF0 (*t*_0_ = 1 mm, *l*_0_ = 10 mm); (**a2**) CCF1 (*h* = 1 mm); (**a3**) CCF2 (*h* = 2 mm); (**a4**) CCF3 (*h* = 3 mm); (**b**) Schematic diagram of the CCF samples constructed by 4 × 4 × 4 arrays of the cubic unit cell shown in Figure 1b.

Cubic unit cell models of the COF and the CCF are depicted in Figure 1a,b, respectively. $t_{0}$ and $l_{0}$ are the edge length and edge thickness of the COF, and the CCF have sine-shaped cell edges with chord span $l_{0}$, chord height *h*, and side thickness *t*. Geometrical parameters play a key role for mechanical properties of the foams [45]. To show the effects of geometrical topology on mechanical behaviors, including elastic moduli, energy absorption, and fracture characteristic, of the CCF, here one COF and three kinds of CCF of equal mass are taken as the examples for illustration. As shown in Figure 2a, the four kinds of samples are named COF0, CCF1, CCF2, and CCF3, respectively. They are constructed by 4 × 4 × 4 arrays (Figure 2b) of their cubic unit cell (Figure 1a,b). The materials of the samples are VeroWhite Plus, whose density and Poisson’s ratio are 1.18 kg/m^3^ and 0.33.

Here, the edge length and edge thickness of the COF0 are *t*_0_ = 1 mm, *l*_0_ = 10 mm. The chord height *h* of CCF1, CCF2, and CCF3 are 1 mm, 2 mm, and 3 mm, respectively (Figure 2a). Apparently, when *h* = 0, the CCF becomes the COF.

The curve of the sine-shaped cell edges (Figure 1c) of the CCF samples can be mathematically described as $y=h\sin\left(\pi /l_{o}x\right)$ ($x\in [0,\;l_{0}]$). To avoid the intersection of the sine-shaped cell edges, here $h/l_{0}<0.5$ is assumed. Define the curve length of the sine-shaped cell edges as *s*, then *s* is expressed as:

(1) $$s=\int_{0}^{l_{0}}\sqrt{1+{\left(y^{\prime }\right)}^{2}}dx=\int_{0}^{l_{0}}\sqrt{1+h^{2}\pi^{2}/l_{0}^{2}\cos^{2}\left(\pi /l_{0}x\right)}dx\;$$

According to the equal-mass principle, we have $st=t_{0}l_{0}$, which gives the side thickness *t* of the sine-shaped cell edges of the CCF samples

(2) $$t=t_{0}l_{0}/\int_{0}^{l_{0}}\sqrt{1+h^{2}\pi^{2}/l_{0}^{2}\cos^{2}\left(\pi /l_{0}x\right)}dx\;$$

Substituting, *t*_0_ = 1 mm, *l*_0_ = 10 mm, *h* = 1,2,3 mm into Equation (2) gives a side thickness *t* of CCF1, CCF2, and CCF3. They are 0.9975, 0.99, and 0.9779, respectively. At the same time, apparently, the chord height to span ratio $h/l_{0}$ of the COF0, CCF1, CCF2, and CCF3 are 0, 0.1, 0.2, and 0.3, respectively.

## 3. Effect of Geometrical Morphology on Elastic Moduli of the NPR CCF

In this part, effects of geometrical topology on elastic moduli, including Poisson’s ratio and relative Young’s modulus, of the COF0 ($h/l_{0}=0$), CCF1 ($h/l_{0}=0.1$), CCF2 ($h/l_{0}=0.2$), and CCF3 ($h/l_{0}=0.3$) are investigated.

**Figure 3:**
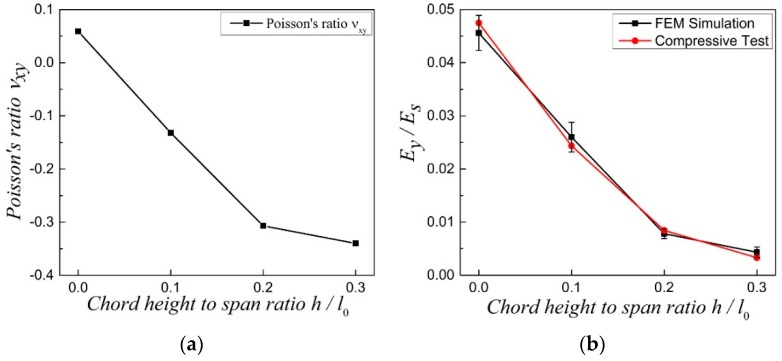
(**a**) Poisson’s ratio $\nu_{xy}$ ($\nu_{xy}=\nu_{yx}=\nu_{xz}=\nu_{zx}=\nu_{yz}=\nu_{zy}$) and (**b**) relative Young’s modulus $E_{y}/E_{s}$ ($E_{x}=E_{y}=E_{z}$) of the COF0, CCF1, CCF2 and CCF3, in which $E_{y}$ is the Young’s modulus of the CCF in the *y* direction and $E_{s}$ is the Young’s modulus of the solid of which the CCF are made of.

Poisson’s ratio $\nu_{xy}$ of the four samples are calculated numerically by exerting periodic boundary conditions [46] on their own cubic unit cell in the orthogonal *x*, *y* and *z* directions. Due to the geometrical symmetry of the COF and CCF, it is not difficult to imagine that $\nu_{xy}=\nu_{yx}=\nu_{xz}=\nu_{zx}=\nu_{yz}=\nu_{zy}$. From Figure 3a, it is easy to see that Poisson’s ratio $\nu_{xy}$ of COF0, CCF1, CCF2, and CCF3 are 0.06, −0.13, −0.31, and −0.35, respectively. Apparently, the COF, whose cell edges are straight, have PPR, but the CCF, whose cell edges are sine-shaped curved, have NPR and with the increase of the chord height to span ratio $h/l_{0}$ of the CCF, the effect of NPR accelerates. In other words, with the cell edges changing from straight shape (COF) to curved sinoidal shape (CCF), Poisson’s ratios transform from positive to negative and in some extent, the more curved the cell edges of the CCF, the more obvious the effect of NPR. This indicates that the NPR of the CCF can be tuned through changing the degree of curvature, i.e., the chord height to span ratio $h/l_{0}$, of the CCF.

As shown in Figure 3b, relative Young’s modulus $E_{y}/E_{s}$ of the four samples are investigated through the numerical and experimental methods. Here, $E_{y}$ is the Young’s modulus of the CCF in the *y* direction and $E_{s}=2500\;\mathrm{MPa}$ is the Young’s modulus of the solid of which the CCF are made of. Due to the geometrical symmetry of the COF and CCF, it is also not difficult to imagine that $E_{x}=E_{y}=E_{z}$. Like the Poisson’s ratio $\nu_{xy}$, $E_{y}$ of the four samples by numerical simulations are also calculated through exerting periodic boundary conditions on their own cubic unit cell in the orthogonal *x*, *y*, and *z* directions [46]. On the other hand, $E_{y}$ by the experiments are obtained from the linear elastic stage of quasi-static uniaxial compressive tests, the details of which will be discussed in Section 4. From Figure 3b, we can see that, as a whole, the relative Young’s modulus $E_{y}/E_{s}$, as calculated by numerical simulations, agree well with that of the experiments. The relative Young’s modulus $E_{y}/E_{s}$ decreases with the increase of the chord height to span ratio $h/l_{0}$ of the cell edges. In other words, stiffness of the studied four kinds of samples decreases with the increase of the chord height to span ratio.

**Figure 4:**
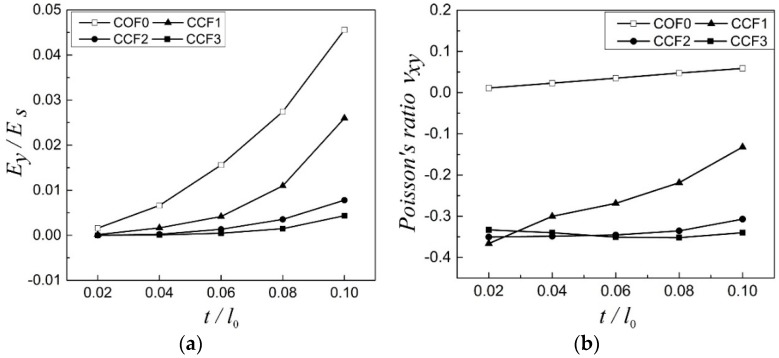
(**a**) The relative Young’s modulus $E_{y}/E_{s}$ and (**b**) Poisson’s ratio $\nu_{xy}$ vs. $t/l_{0}$ for $h/l_{0}=0,\;0.1,\;0.2,\;0.3$.

Except the examples of COF0, CCF1, CCF2, and CCF3, to further investigate the effect of geometrical morphology on elastic moduli of the CCF, the following parameters are taken as examples for more illustration: $h/l_{0}=0,\;0.1,\;0.2,\;0.3$, $t/l_{0}=0.02:0.02:0.1$. The relative Young’s modulus $E_{y}/E_{s}$ and Poisson’s ratio $\nu_{xy}$ vs. $t/l_{0}$ are shown in Figure 4a,b, respectively. From Figure 4a, it is easy to see that for the fixed $h/l_{0}$, the relative Young’s modulus $E_{y}/E_{s}$ increases with the increase of $t/l_{0}$. For the fixed $t/l_{0}$, as a whole, the relative Young’s modulus $E_{y}/E_{s}$ decreases with the increase of $h/l_{0}$. With respect to the Poisson’s ratio (Figure 4b), for $h/l_{0}=0$, $\nu_{xy}$ is positive and it slightly increases with the increase of $t/l_{0}$. For $h/l_{0}=0.1$, $\nu_{xy}$ is negative and the effect of NPR decreases obviously with the increase of $t/l_{0}$. For $h/l_{0}=0.2$, $\nu_{xy}$ is negative and keeps almost a constant when $t/l_{0}$ increases from 0.02 to 0.06. Then, the effect of NPR slightly decreases when $t/l_{0}$ increases from 0.06 to 0.1. For $h/l_{0}=0.3$, $\nu_{xy}$ is negative and almost keeps a constant with the increase of $t/l_{0}$. For the fixed $t/l_{0}$, in general, the effect of NPR of the CCF increases with the increase of $h/l_{0}$.

## 4. Effect of Geometrical Morphology on Energy Absorption Properties the NPR CCF

To show the effect of geometrical morphology on energy absorptions of the CCF, uniaxial quasi-static compression tests (MTS E46 (MTS Systems Corporation, Eden Prairie, MN, USA) with a loading rate of 1 mm/min) are performed on the four kinds of 3D-printed samples COF0, CCF1, CCF2, and CCF3. For each kind of sample, the experiments are repeated three times. Specific energy absorption (SEA) [47,48,49,50,51] is used to evaluate the energy absorption capacity of the samples, which is expressed as

(3) $$\mathrm{SEA}=\frac{\mathrm{EA}}{m}=\frac{\int_{0}^{L}F(x)dx}{m}\;$$

where EA is the energy absorption, *L* is the effective total crushing length (that is to say, the crushing length of the regime of densification, in which the crushing force rises steeply, is not included) [45], and *F* is the crushing force and *m* is the mass of the structure.

**Figure 5:**
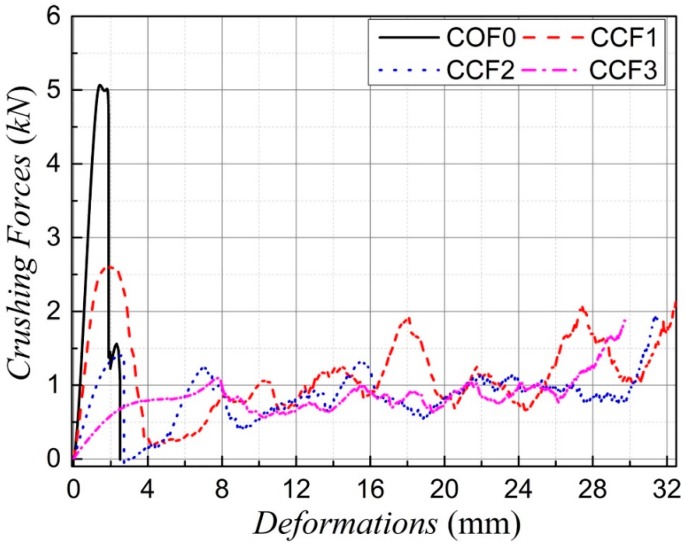
Typical crushing force-deformation curves of the four sample COF0, CCF1, CCF2, and CCF3 under uniaxial quasi-static compression in the *y* direction.

Typical axial load-longitudinal deformation curves of the four samples are given in Figure 5, in which the curves for the NPR CCF1, CCF2, and CCF3 are approximately truncated at the beginning of the final stage of densification where the crushing force begins to rise steeply [45]. It is obvious that under uniaxial quasi-static compression the PPR COF0 is brittle crushing, without the phenomenon of collapse plateau. However, for CCF1, CCF2, and CCF3, the long collapse plateau exists in the loading process, which means that the CCF could have much higher energy absorption capacities than the COF. At the same time, as a whole, crushing forces of CCF1 are larger those of CCF2 and CCF3. It means that energy absorption capacity of the CCF1, whose NPR effect is weaker than CCF2 and CCF3, is larger than those of CCF2 and CCF3.

**Figure 6:**
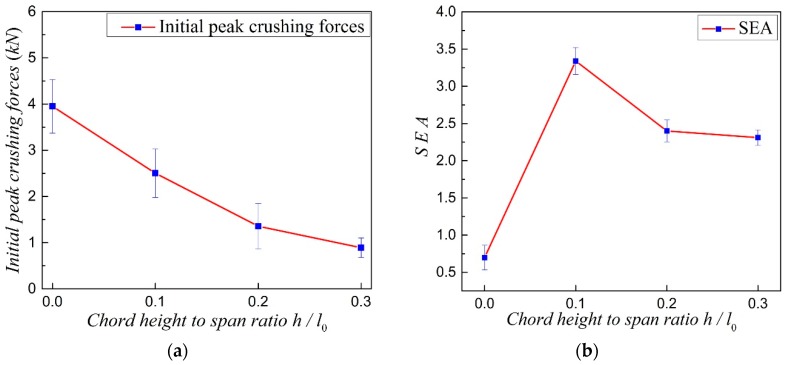
(**a**) Initial peak crushing forces and (**b**) specific energy absorption (SEA) of the three-dimensional (3D)-printed four samples COF0, CCF1, CCF2, and CCF3.

Initial peak crushing forces and SEA of the four samples are shown in Figure 6a,b, respectively. From Figure 6a, it is easy to see that the initial peak crushing forces, i.e., the strengths, of the four samples decrease with the increase of the chord height to span ratio. Figure 6b shows that the SEA of NPR CCF1, CCF2, and CCF3 are much larger than that of the PPR COF0, and SEA of CCF1, whose NPR effect is weaker than CCF2 and CCF3, is larger than those of the CCF2 and CCF3. From the above mentioned analysis, it can come to the conclusion that energy absorption capacity of the CCF can be tuned and enhanced through the rational design of the chord height to span ration, i.e., the geometrical morphology or NPR, of the CCF. In other words, by slightly introducing the effect of NPR, the energy absorption abilities of the CCF could be greatly enhanced with only slightly reducing the stiffness and strength of the CCF.

## 5. Effect of Geometrical Morphology on Collapse Modes of the NPR CCF under Quasi-Static Uniaxial Compressions

In this part, effect of geometrical morphology on collapse modes of the NPR CCF under quasi-static uniaxial compressions are investigated.

**Figure 7:**
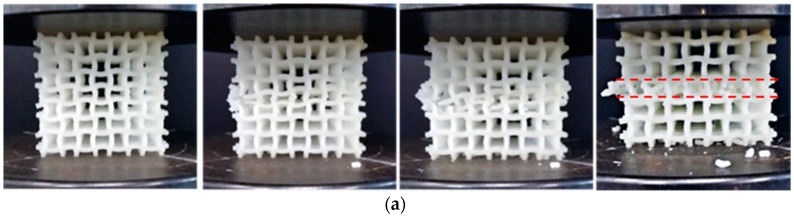

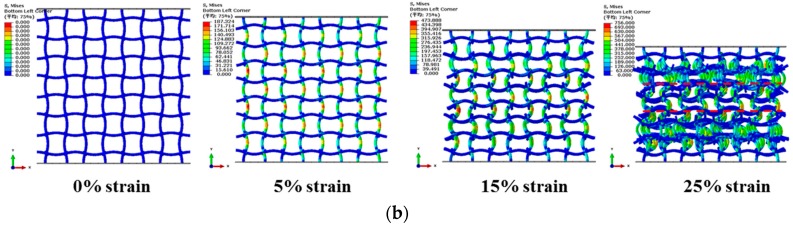
Front view of collapse modes of the NPR CCF1 under quasi-static uniaxial compressions in the *y* direction: (**a**) experiment; (**b**) numerical simulation (until 25% strain).

**Figure 8:**
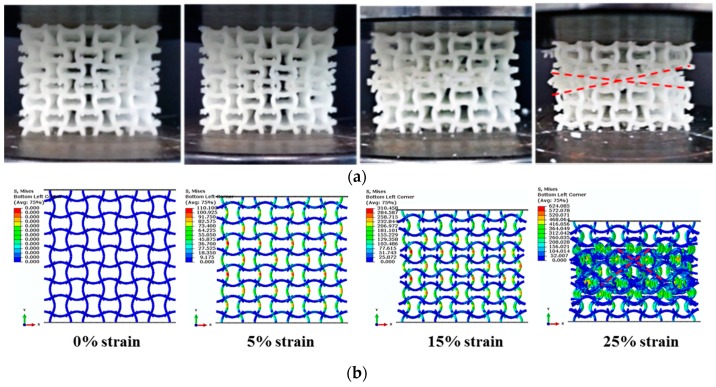
Front view of collapse modes of the NPR CCF2 under quasi-static uniaxial compressions in the *y* direction: (**a**) experiment; (**b**) numerical simulation (until 25% strain).

**Figure 9:**
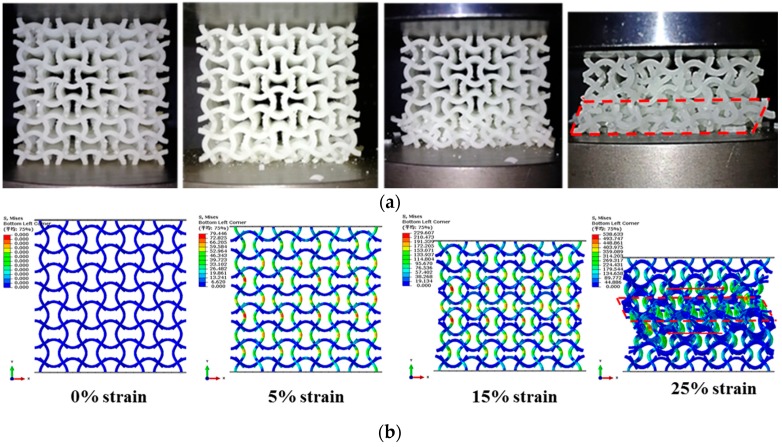
Front view of collapse modes of the NPR CCF3 under quasi-static uniaxial compressions in the *y* direction: (**a**) experiment; (**b**) numerical simulation (until 25% strain).

Under quasi-static uniaxial compressions, collapse mode of the PPR COF0 is abrupt layer by layer brittle fracture in a very short time. It is very difficult to take photos of the collapse process using the common camera. So, here, collapse modes of the PPR COF0 are not discussed. Typical collapse modes of the NPR CCF1, CCF2 and CCF3 under uniaxial quasi-static compressive tests (Figure 5) are shown in Figure 7a, Figure 8a and Figure 9a, respectively. Numerical simulations of collapse process until 25% strain of the NPR CCF1, CCF2, and CCF3 under quasi-static compressions in the *y* direction are shown in Figure 7b, Figure 8b and Figure 9b, respectively. Details of the numerical simulations are as follows: two rigid plates with size 40 mm × 40 mm × 2 mm, elastic modulus 210,000 MPa, and Poisson’s ratio 0.33, have been put on the top and bottom of the COF or CCF along the *y* direction. Elastic modulus and Poisson’s ratio of the materials, of which the COF and CCF are made of, are 2500 MPa and 0.33, respectively. The rigid plates are connected with the COF or CCF using the tie constrains. The bottom rigid plate is fixed. An uniaxial displacement of 10 mm is exerted on the top rigid plate in the *y* direction. The COF and CCF are constituted by 64 cubic unit cells (Figure 1a,b). B31 beam element is used for the COF and CCF. The beam element length is 0.1 mm and the number of the beam elements is 76,800. For the top and bottom rigid plates, the C3D8R element is utilized. The length of the element is 0.5 mm and the corresponding elements number for each rigid plate is 400. In addition, the default self-contact boundary conditions given by Abaqus have been used to deal with the contact interactions between the cell edges.

As a whole, experimental results and numerical simulations of collapse modes of the NPR CCF1 (Figure 7) agree very well with each other, as well as the NPR CCF2 (Figure 8). Experimental results (Figure 9a) and numerical simulations (Figure 9b) of the collapse modes of the NPR CCF3 are different. Due to the fabrication errors produced in the 3D-printed process, in the experiment (Figure 9a), the initial collapse starts from the bottom end of the CCF3. In the numerical simulation (Figure 9b), the initial collapse starts from the middle layer of the CCF3. But, their final collapse modes (Figure 9a,b) are similar.

From Figure 7, Figure 8 and Figure 9, it is obvious that under uniaxial compression in the *y* direction, the CCF1, CCF2, and CCF3 all shrink in the transverse *x* direction, indicating their NPR characteristics. However, their collapse modes are different. For the CCF1 (Figure 7) whose NPR effect is relatively weak, in the uniaxial quasi-static compressive loading process, stresses are mainly concentrated on the convex and concave parts of the vertical curved cell edges (Figure 7a,b), so it collapses layer by layer from the peak points of the convex and concave vertical cell edges. For the CCF2 (Figure 8), whose cell edges are more curved than CCF1 (i.e., the NPR effect is more obvious than CCF1), under uniaxial quasi-static compressions, the adjacent curved cell edges will contact with each other. It renders a collapse mode of ‘X’ shape for CCF2, as that shown in Figure 8a,b. For the CCF3 (Figure 9), whose cell edges are much more curved ($h/l_{0}=0.3$), under compressions the adjacent curved cell edges will be more easier to contact with each other and the horizontal slipping shears occur in the middle layers. Finally, it exhibits the oblique quadrilateral collapse mode for CCF3 (Figure 9a,b).

## 6. Effect of Geometrical Morphology on Damage Properties of the NPR CCF

In this part, effect of geometrical morphology on damage properties of the NPR CCF is investigated through the plastic-damage constitute model that was proposed by Senaz [52], which is initially developed for concrete.

### 6.1. Plastic-Damage Constitute Model for the 3D-Printed VeroWhite Plus Materials

First, plastic-damage constitute model for the 3D-printed VeroWhite Plus materials is introduced. It is well known that for the heterogeneous materials there is an important relation between the damage field and deformations of the microstructures [53,54,55,56]. Increase of the damage means the deterioration of the structural integrity of the intrinsic microstructures. Due to the heterogeneous characteristics of the 3D-printed VeroWhite Plus materials, damage distributions of the NPR CCF could be studied through the plastic-damage constitute model [53,57,58,59,60], thus predicting and explaining macro deformations and fracture behaviors of the NPR CCF.

**Figure 10:**
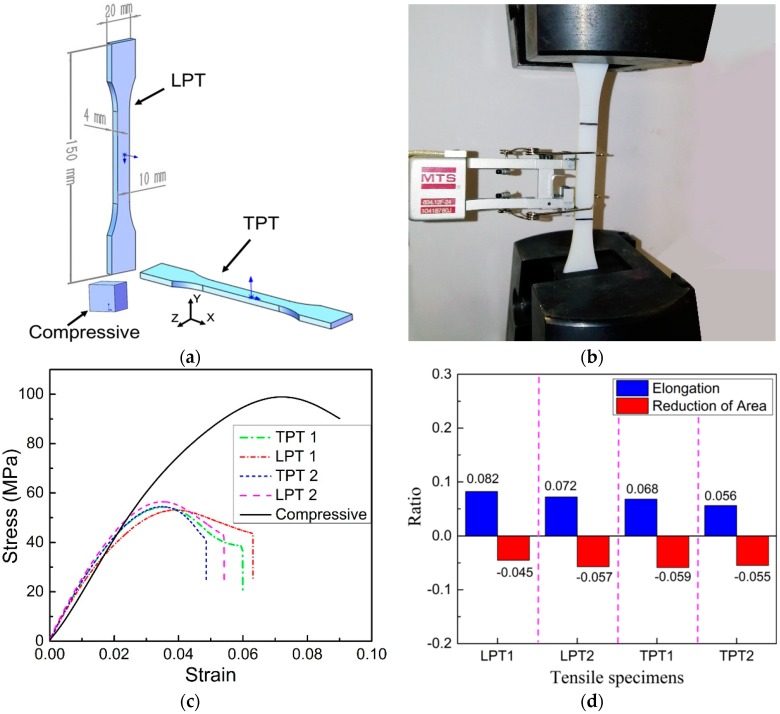
(**a**) 3D-printed VeroWhite specimens for tensile and compressive tests: transversely printed tensile specimen (TPT) means transversely printed tensile dog-bone shaped specimens, longitudinally printed tensile specimens (LPT) means longitudinally printed tensile dog-bone shaped specimens and the cubic specimen is for compressive test; (**b**) Tensile tests of the VeroWhite TPT and LPT specimens; (**c**) Stress-strain curves of the 3D-printed VeroWhite Plus TPT, LPT, and compressive cubic specimens; (**d**) Ratios of elongation and reduction of area of the TPT and LPT.

To establish the plastic-damage constitute model for the 3D-printed VeroWhite Plus materials, tensile and compressive tests are performed on the 3D-printed VeroWhite Plus specimens (Figure 10a). Tensile tests (Figure 10a,b) are performed on two kinds of dog-bone shaped specimens, one kind is the transversely printed tensile specimen (TPT) and the other kind is the longitudinally printed tensile specimens (LPT). Tensile tests, according to the ASTM D638 standard [61], of each kind of specimens are repeated two times on MTS E46 with a loading rate of 2 mm/min. The compressive test is performed on the cubic specimen with size 1 mm × 1 mm × 1 mm (Figure 10a). The corresponding stress-strain curves of the tensile and compressive tests are shown in Figure 10c.

Compressive stress-strain curve (Figure 10c) of the 3D-printed VeroWhite Plus materials is similar to that of the pure concrete, so the plastic-damage model, proposed by Senaz [52], for pure concrete could be used for the compressive damage analysis of the 3D-printed VeroWhite Plus materials. The plastic-damage model could also be used for tensile damage analysis of the 3D-printed VeroWhite Plus materials. The reasons are that the ratios of elongation and reduction of area of the 3D-printed VeroWhite Plus TPT and LPT are very small (Figure 10d), and, at the same time, tensile stress-strain curves of the TPT and LTP are similar to that of the compressive test, which also show obvious linear elastic stage and plastic damage stage. From the above analysis, the plastic-damage model that was proposed by Senaz [52], could be used for both the compressive and tensile damage analysis of the 3D-printed VeroWhite Plus materials.

In the linear elastic stage, the relation between the stress and the strain is expressed as:

(4) $$\sigma \;=\;E_{0}\varepsilon \;$$

In the plastic damage stage it is given by

(5) $$\sigma \;=\;\frac{E_{0}\varepsilon }{1+(\alpha +\frac{E_{0}}{E_{s}}-2)(\frac{\varepsilon }{\varepsilon_{0}})+(1-2\alpha ){\left(\frac{\varepsilon }{\varepsilon_{0}}\right)}^{2}+\alpha {\left(\frac{\varepsilon }{\varepsilon_{0}}\right)}^{3}}\;$$

in which *σ* is the stress, *ε* is the strain, $E_{0}$ is the initial elastic modulus, $\varepsilon_{0}$ is the strain corresponding to the peak stress $\sigma_{0}$, $E_{s}$ is the secant modulus, and *α* is the characteristic coefficient which has the form

(6) $$\alpha \;=\;\frac{\frac{E}{E_{0}}(\frac{\sigma_{0}}{\sigma_{u}}-1)}{{\left(\frac{\varepsilon_{u}}{\varepsilon_{0}}-1\right)}^{2}}-\frac{\varepsilon_{u}}{\varepsilon_{0}}\;$$

Here, $\sigma_{u}$ is the ultimate stress and $\varepsilon_{u}$ is the strain corresponding to $\sigma_{u}$ [52].

The total damage *D*, summation of the compressive and tensile damages, is expressed as:

(7) $$D\;=\;1-\sqrt{\frac{\sigma }{E_{0}\varepsilon }}\;$$

Substituting Equations (5) and (6) into Equation (7) gives the formula of damage evolution for the 3D-printed VeroWhite Plus materials

(8) $$D\;=\;\{\begin{array}{ll} 0 & \mathrm{Elastic}\;\mathrm{stage}\\ 1-\frac{1}{\sqrt{1+(\alpha +\frac{E_{0}}{E_{S}}-2)(\frac{\varepsilon }{\varepsilon_{0}})+(1-2\alpha )(\frac{\varepsilon }{\varepsilon_{0}})+\alpha (\frac{\varepsilon }{\varepsilon_{0}})}} & \mathrm{Plastic}\;\mathrm{damage}\;\mathrm{stage} \end{array}\;$$

**Figure 11:**
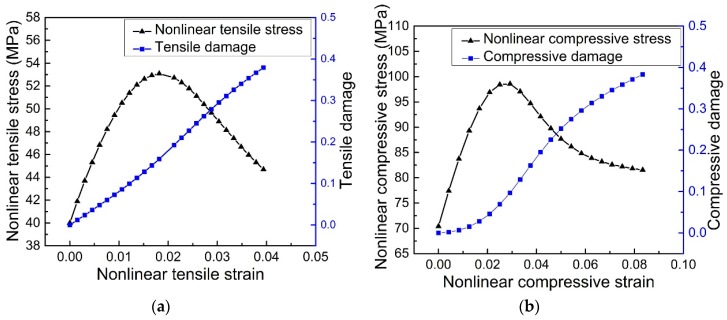

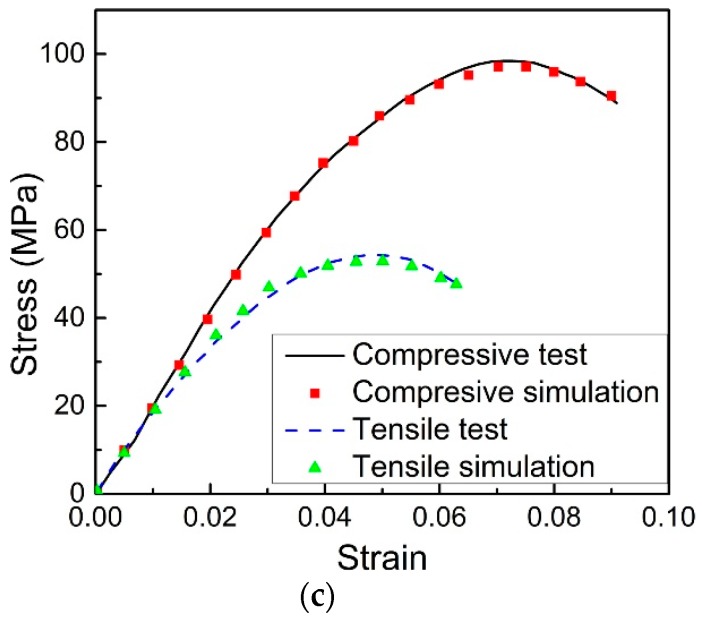
(**a**) Tensile and (**b**) compressive plastic-damage parameters of the 3D-printed VeroWhite Plus materials; (**c**) Comparison of stress-strain curves of the tensile dog-bone shaped specimen and compressive cubic specimen: experiments and numerical simulations.

In Section 6.2, damage properties of the 3D-printed VeroWhite Plus NPR CCF will be numerically investigated through Abaqus. The corresponding tensile and compressive plastic-damage parameters for the numerical simulations are shown in Figure 11a,b, respectively. These parameters are calculated by combining the stress-strain curves, of the 3D-printed VeroWhite Plus TPT, LPT and compressive cubic specimens (Figure 10c), and Equation (7). To verify these plastic-damage parameters, the stress-strain curves of the tensile dog-bone shaped specimen and compressive cubic specimen are numerically simulated. The related numerical results and the experimental results are shown in Figure 11c. Obviously, they agree very well with each other, indicating that the damage properties of the NPR CCF could be analyzed using the tensile and compressive plastic-damage parameters shown in Figure 11a,b.

### 6.2. Damage Properties of the NPR CCF

Utilizing the periodic boundary conditions and simply choosing 2 × 2 × 2 arrays of the cubic unit cell (Figure 1b) as the model, damage properties of the COF0, CCF1, CCF2, and CCF3 under uniaxial compressive loadings in the *y* direction are numerically studied through the plastic-damage constitute model that is given in Figure 11a,b.

**Figure 12:**
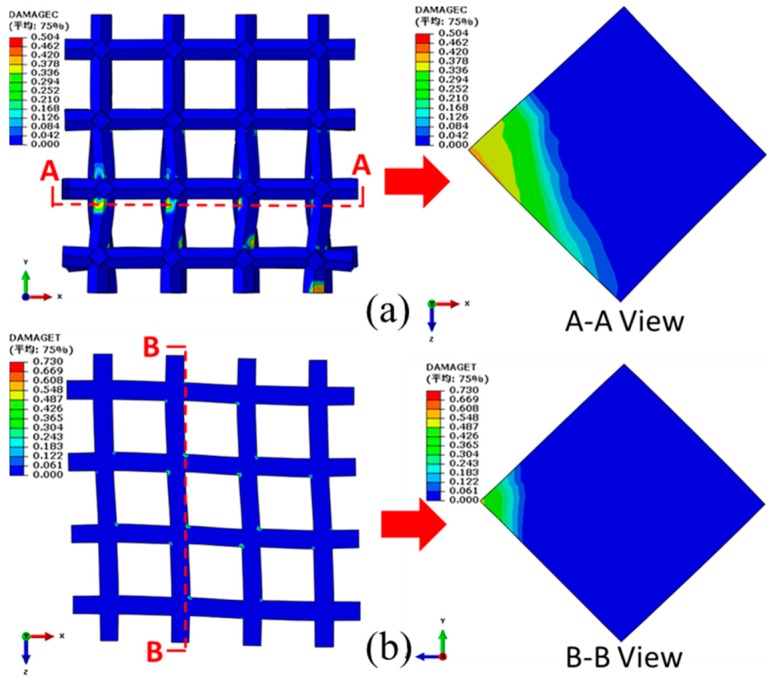
Damage distributions of the COF0 under the compressive displacement of 0.75 mm (uniaxial strain of 3.75%) in the *y* direction: (**a**) compressive damages; (**b**) tensile damages.

Fixing the bottom end, a compressive displacement of 0.75 mm (uniaxial strain of 3.75%) is exerted on the top of the COF0 in the *y* direction. The corresponding compressive and tensile damage distributions (front view) of the COF0 are shown in Figure 12a,b, respectively. From Figure 12a, it is easy to see that severe buckling deformations occur at the intersections between the vertical cell edges and the horizontal cell edges. From the view of A-A crosscuttings, cross sections of the vertical cell edges show mainly compressive damage and almost no tensile damage. From the view of B-B crosscuttings, tensile damage occurs near the intersections of cross sections of the horizontal cell edges.

**Figure 13:**
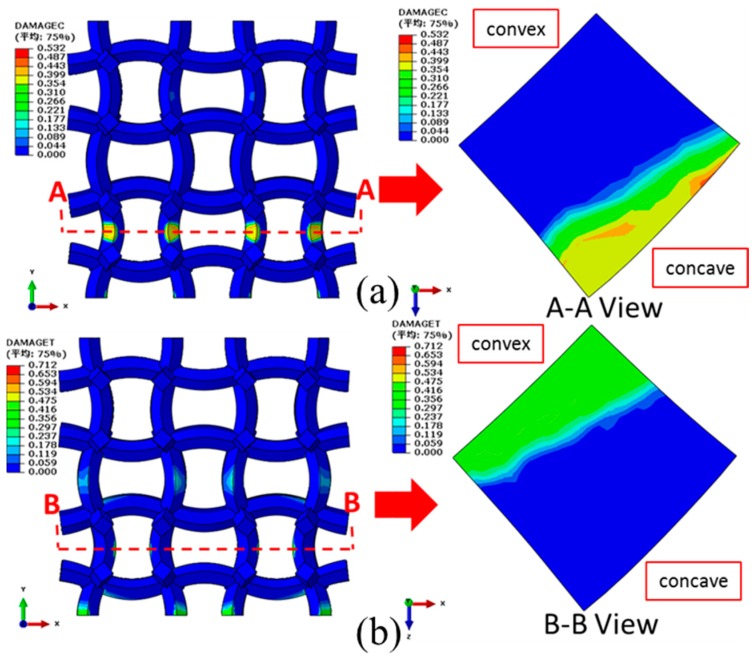
Damage distributions of the CCF1 under the compressive displacement of 1 mm (uniaxial strain of 5%) in the *y* direction: (**a**) compressive damages; (**b**) tensile damages.

Likewise, a compressive displacements of 1 mm (uniaxial strain of 5%) is exerted on top of the CCF1 in the *y* direction. As shown in Figure 13a, compressive damages are concentrated on the inner concave parts of the vertical curved cell edges. As shown in Figure 13b, tensile damages are concentrated on the outer convex parts of the vertical curved cell edges. Almost no damage occurs for the horizontal curved cell edges. The maximum damages occur on edges of cross sections of peak points of the inner concave (view of AA crosscutting in Figure 13a) and outer convex (view of BB crosscutting in Figure 13b) parts, which will firstly lead to the initial fracture of the structures.

**Figure 14:**
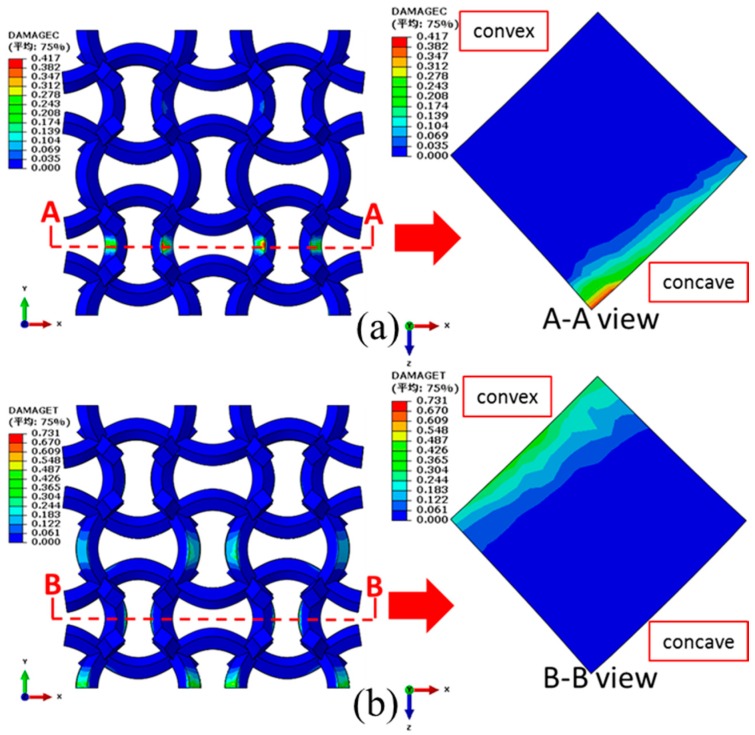
Damage distributions of the CCF2 under the compressive displacement of 1.5 mm (uniaxial strain of 7.5%) in the *y* direction: (**a**) compressive damages; (**b**) tensile damages.

Compared with the CCF1, a larger compressive displacement of 1.5 mm (uniaxial strain of 7.5%) is exerted on CCF2 in the *y* direction. However, the compressive (Figure 14a) and the tensile (Figure 14b) damages of the CCF2 are obviously reduced. From the views of AA (Figure 14a) and BB (Figure 14b) crosscuttings, it is also apparent that both the area and the peak value of the compressive and tensile damages are decreased. These indicate that the introducing of NPR could enhance the damage resistance capacity of the CCF.

**Figure 15:**
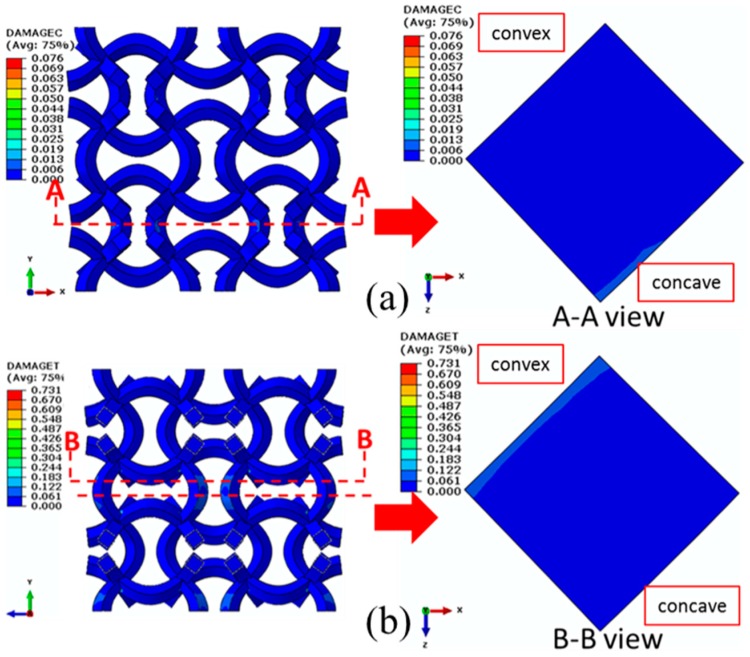
Damage distributions of the CCF3 under the compressive displacement of 1.65 mm (uniaxial strain of 8.25%) in the *y* direction: (**a**) compressive damages; (**b**) tensile damages.

When compared with the CCF2, a much larger compressive displacement of 1.65 mm (uniaxial strain of 8.25%) is exerted on CCF3 in the *y* direction. Apparently, compressive (Figure 15a) and tensile (Figure 15b) damages of CCF3 are much more reduced, indicating that damage resistance capacity of the CCF3 is much more enhanced. From the view of AA crosscutting, the maximum compressive damage still occurs on edges of cross sections of peak points of the inner concave. But, the maximum tensile damage deviates a distance from the edges and is not on edges of cross sections of peak points of the outer convex anymore.

**Figure 16:**
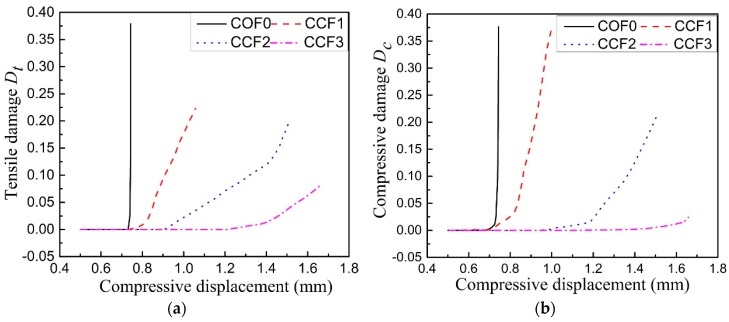
The maximum damages of the cross sections of the vertically placed cell edges under different compressive displacements: (**a**) compressive damages; (**b**) tensile damages.

To further explain the effect of geometrical morphology on damage properties of CCF, the maximum damages of the cross sections of the vertically placed cell edges under different compressive displacements have been calculated. The corresponding maximum compressive and tensile damages versus the compressive displacements are given in Figure 16a,b, respectively. It is obvious that the increase of the effect of NPR could postpone the occurrence of structural damage of the CCF and it decrease the damage degree.

## 7. Conclusions

The orthogonal isotropic NPR CCF are a typical kind of mechanical metamaterials, which have great potential applications in the fields of mechanical and aerospace engineering. Systematic and deep understanding of their mechanical properties plays a vital role for their practical engineering applications. In this paper, based on the polymer materials VeroWhite Plus and mainly choosing one COF and three kinds of CCF of equal mass as examples, the effects of the geometrical morphology on elastic moduli, energy absorption, and damage characteristics of the CCF, are systematically investigated through the experimental and finite element methods. Results show that NPR, energy absorption, and damage properties of the CCF could be tuned simultaneously through adjusting the chord height to span ratio of the sine-shaped cell edges. By rational design of the NPR, as compared to the COF of equal mass, the CCF could have combined advantages of relative high stiffness and strength, enhanced energy absorption, and damage resistance. The study of this paper provides the theoretical foundations for optimization design of mechanical properties of the NPR CCF and thus could promote their practical applications in the engineering fields. However, one thing noteworthy is that this paper has only focused on mechanical properties of the polymer (VeroWhite Plus) CCF, due to their easy fabrication through the 3D-printed method. In fact, the stiffness and strength of the VeroWhite Plus materials are very low. For further enlarging, their practical engineering applications, the method to make much stiffer and higher strength metal CCF should be explored.
